# Supplementary material for: The causal relationship between the human gut microbiota and pyogenic arthritis: a Mendelian randomization study
Source: Front Cell Infect Microbiol. 2024 Nov 26;14:1452480. doi: 10.3389/fcimb.2024.1452480 (PMC11629706; doi:10.3389/fcimb.2024.1452480)
Supplement: Supplementary file 1 [file DataSheet1.zip › mendelian test/ebi-a-GCST90027706.csv_forest.pdf]

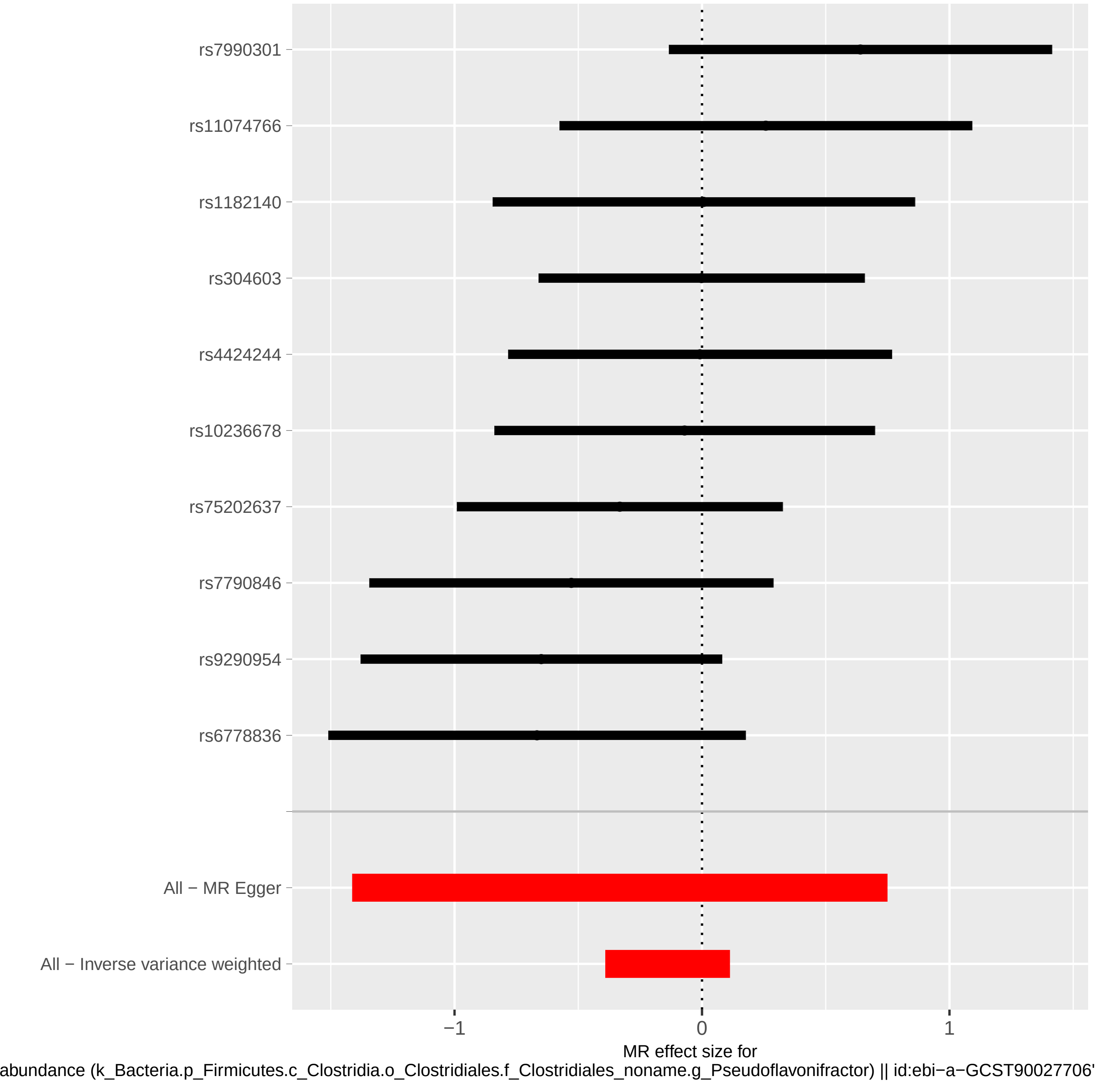
rs7990301
rs11074766
rs1182140
rs304603
rs4424244
rs10236678
rs75202637
rs7790846
rs9290954
rs6778836
All − MR Egger
All − Inverse variance weighted
−1
0
1
MR effect size for
abundance (k_Bacteria.p_Firmicutes.c_Clostridia.o_Clostridiales.f_Clostridiales_noname.g_Pseudoflavonifractor) || id:ebi−a−GCST90027706'
